# Evidence of variable bacterial colonization on coloured elastomeric ligatures during orthodontic treatment: An intermodular comparative study

**DOI:** 10.4317/jced.54610

**Published:** 2018-03-01

**Authors:** Ravish Sharma, Kavita Sharma, Rajesh Sawhney

**Affiliations:** 1 Department of Orthodontics, Bhojia Dental College & Hospital, Bhud (Baddi); 2 Private Practice, Panchkula (Haryana) India; 3 Professor & Head, Department of Microbiology, Rayat-Bahra Dental College & Hospital, Sahauran, Mohali (Pb.) India

## Abstract

**Background:**

Besides, other factors, the choice of materials used as orthodontic ligatures could be one of the many tools to counter the effects of microbial adhesion, that culminates into dental ailments. Therefore, we assessed bacterial adhesion on elastomeric ligatures with special reference to coloured elastomeric rings during orthodontic treatment.

**Material and Methods:**

A split mouth study, involving 240 samples of different elastomeric ligatures from forty orthodontic patients possessing good oral hygiene was carried out. The archwire was ligated to the brackets on both arches with elastomeric rings (superslick, clear transparent , blue and pink) at predetermined quadrants. After six weeks, ligatures from second premolars were removed and processed for bacterial enumeration using standard techniques. Bacterial counts were also determined for stimulated saliva samples taken at 0 and 6 weeks.

**Results:**

A statistically significant difference in bacterial counts was obtained amongst different elastomeric modules used. Maximum bacterial counts were found on conventional pigmented elastomeric modules, followed by Superslick module and clear module. More number of bacteria associated with the conventional pink as compared to the conventional blue pigmented modules, however it was not statistically significant. The three bacterial genera *Streptococcus Staphylococcus* and *Aerobic Lactobacilli* adhered to elastomeric modules in following predominant pattern i.e. Conventional pink>Conventional Blue>Superslick>Clear.

**Conclusions:**

The studies evidenced colour and material dependent bacterial colonization on orthodontic modules and could be an indicator of bacterial biofilm forming potential based on surface chemistries and a clinically efficacious tool to redesign conventional and modified elastomeric rings as orthodontic ligation accessories.

** Key words:**Bacterial colonization, biofilm, coloured elastomers, orthodontic ligatures.

## Introduction

Oral cavity is a biologically complex ecosystem with a vast and diverse collection of microorganisms despite the presence of a complex array of components of host defenses. The ecological stress referred to as a shift of the microbiological balance creates conditions conducive to the growth and appearance of cariogenic and periodontopathic bacteria (1).

Patients receiving orthodontic treatment have altered environment in the oral cavity with respect to drop in pH and creation of additional retentive sites for food and bacterial flora like *Streptococcus mutans*, *Lactobacilli* etc. and tend to increase the levels of microorganisms in saliva and in dental biofilm (2-5). Despite the advances in orthodontic materials and treatment mechanics, the placement of fixed appliances is still associated with a high risk of developing white spot lesions, which is considered to be a precursor of frank enamel caries and in orthodontics has been attributed to the prolonged accumulation and retention of bacterial plaque on the enamel surface adjacent to appliances (6).

Patients often have difficulty maintaining adequate oral hygiene with orthodontic appliances attached directly to the teeth. The increased plaque accumulation and the concomitant bacterial acid production result in decalcification by diffusion of calcium and phosphate ions out of enamel. The enamel demineralization is caused by organic acids produced mainly by *S. mutans*, which have been shown to be the prime causative organisms of dental caries.

The increased prevalence of enamel decalcification during fixed appliance therapy is partially due to the designs and the irregular surfaces of brackets, bands, wires and other auxillaries, which create stagnation areas for plaque, render tooth cleaning more difficult, and limit naturally occurring self-cleaning mechanisms, such as the movement of the oral musculature and saliva.

Besides other factors the choice of material used as orthodontic ligatures could be one of many tools to counter the effects of microbial adhesion culminating into dental ailments. The studies have documented plaque formation with higher number of microorganisms on elastomeric rings used as orthodontic ligatures as compared to steel wires (2,4). Further, the advent of Metafix Technology using soluble Hydrogel polymer in orthodontics has led to the introduction of super slick elastomeric rings which might reduce the bacterial CFU and in turn reduce enamel demineralisation during the orthodontic treatment (2,5,7,8). The shift to such a choice could obviously limit the usage with in the given economical constraints. However, there is hardly any available authentic data on bacterial adhesion to colored elastomeric rings. Thus, the present studies attempt to assess inter modular differences in bacterial load on different elastomeric ligatures and address coloured chemistries dependent adhesion on orthodontic ligatures as a valuable tool for choice with in the conventional ligatures.

## Material and Methods

The Prospective Split mouth four quadrant study was carried out in the Department of Orthodontics and Dentofacial Orthopedics at Bhojia Dental college and Hospital, Bhud, Baddi. Distt. Solan, Himachal Pradesh, India. The study population comprised of 240 samples from 40 patients, aged 12-20 years, irrespective of gender who were already undergoing orthodontic treatment in the department. A thorough intra oral and extra oral examination of all the patients was carried out before inclusion in the study following routine oral hygiene instructions and ethical clearance. Patients with active periodontal disease, high caries index, missing /extracted second premolars and any systemic disease were excluded from study.

Archwire Ligation and collection of samples: During the first appointment, after a thorough intra oral and extra oral examination, 3 ml., paraffin wax stimulated, saliva was collected aseptically in sterile container from each patient and processed for bacterial quantification. The patient was asked to refrain from eating or drinking beverages for at least one hour before any oral examination or manipulation so as not to disrupt the oral microbiota.

**Figure 1:**
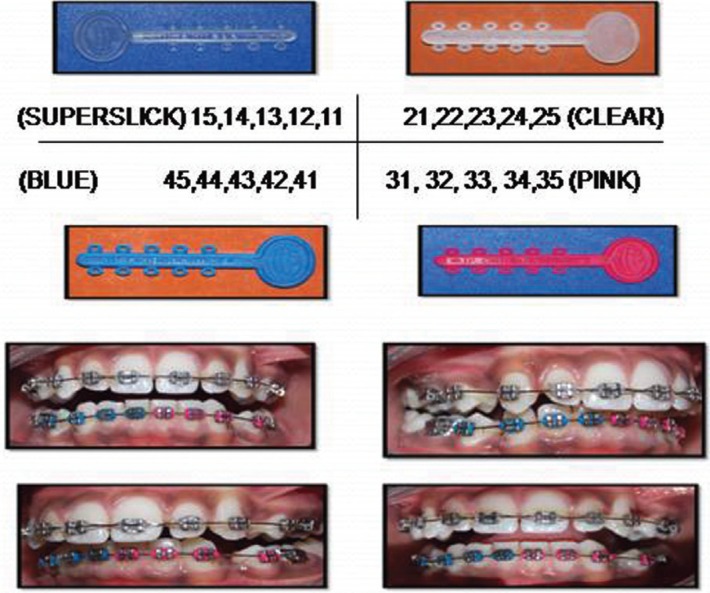
Elastomeric rings and their placement in four quadrants.

The archwire was ligated to brackets on both the arches with the sterile superslick, clear transparent and pigmented conventional blue and pink elastomeric rings from TP Orthodontics Inc la porte, size 120” (Fig. 1) in the predetermined sequence. After a period of six weeks, again the stimulated saliva samples of the patients were collected in a sterile container and subsequently, the elastomeric modules from the second premolars of all the four quadrants were retrieved aseptically, with a sterile probe and tweezer, and collected in pre labelled sterile vials containing transport media. Both saliva and module samples were processed for bacterial quantification and scanning electron microscopic (SEM) studies.

Bacteriological analysis of saliva and module specimen: The bacterial counts were determined using standard method (9). The samples of saliva and modules were suspended separately in sterile 0.01mol phosphate buffered saline solution ( pH 7.4). 10 fold serial dilutions of the saliva and modular suspension were prepared in separate sets up to maximum of 10-8 dilution. 100 ul each of sample suspensions were inoculated separately on Nutrient Agar, Blood Agar and Rogosas Agar plates. The plates were incubated under aerobic conditions at 370C for 24 to 48 hours. The bacterial colonies on the nutrient Agar plates were counted to calculate CFU/ml. The predominant bacterial colonies on blood agar and Rogosas agar were identified based on colony morphology, Gram staining and biochemical tests.

Scanning Electron Microsopy (SEM) of modules: SEM study of modules was done to assess the bacterial colonization on modules using ZEISS EVO Series Scanning Electron Microscope Model EVO 50 by coating the samples with 20-50 nm thick gold or silver with a BIO-RAD POLARAN sputter coater.

Statistical Analysis: Descriptive statistics, including mean, standard deviation, standard error, median, minimum, and maximum values were calculated. The paired t test followed by Wilcoxon signed rank test and Chi test were conducted. The results were considered statistically significant at *p*<0.05. SPSS software (SPSS,Inc,Chicago,III) was used to evaluate the data.

## Results

**Figure 2:**
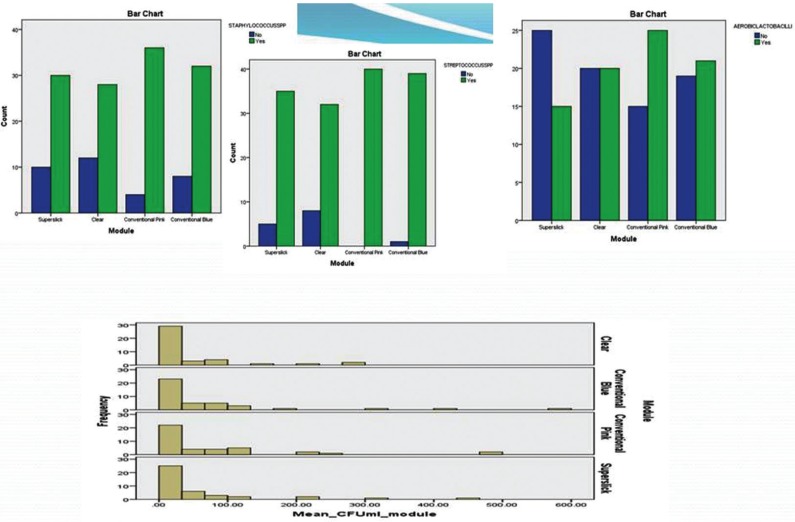
Clustered bar chart of *Staphylococcus*, *Streptococcus* and *Lactobacillus* & Histograms of modules.

The present study showed significantly higher mean bacterial count, approximately 30 times more than the initial level, in saliva after six weeks of orthodontic ligation ( Table 1). Moreover, three different genera with morphological, cultural and biochemical features coinciding with *Streptococcus spp*, *Staphylococcus* and *Aerobic Lactobacilli* were predominantly present in saliva samples. The relative and absolute frequency data showed that all the respondents harbored *Streptococcus spp.* in their saliva at 0 hr and 6 weeks stage. It was noticed that presence of *Staphylococcus spp* was statistically higher in the saliva at six weeks as compared to zero hour stage. There was no statistically significant difference between the presence of Lactobacillus in saliva at two said stages of sampling. The descriptive statistics for the bacterial colonization of four modules showed highest mean bacterial count on conventional pink module (75.5695X 105 CFU/ml ) and the lowest (44.02X105 CFU/ml) for clear module ( Table 2). Similar trend could be seen on plotting line graph for 40 respondents. Since, the spread of the data has been quite high, both the parametric and non-parametric tests were conducted along with histograms (Fig. 2) . The paired sample t-test was also done to study significant difference between the CFU/ml for the various modules ( Table 3). The statistics indicated statistically significant difference in bacterial counts between the Clear and Conventional blue module; Conventional pink and superslick 

**Table 1:**
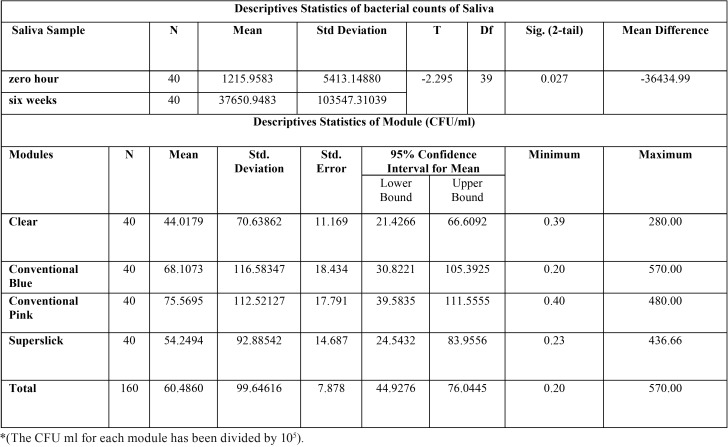
Descriptive statistics for bacterial counts in saliva samples and modules.

**Table 2:**
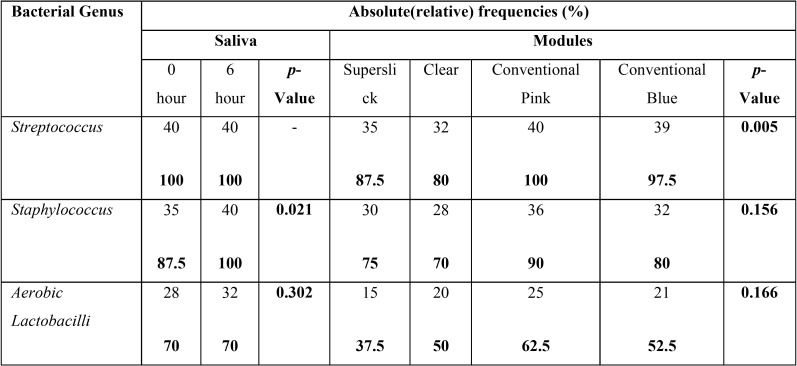
Relative and absolute frequencies of bacteria in Saliva and modules.

**Table 3:**
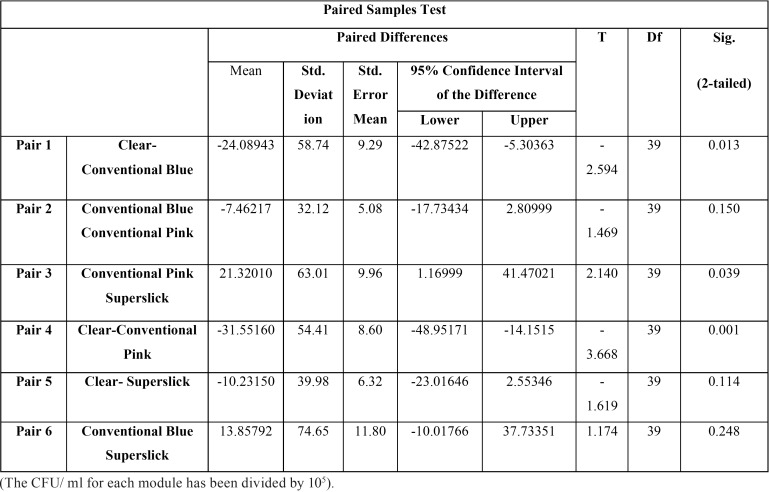
Paired samples test of various modules.

module; Clear and Conventional pink module. Contrary to this, there was not enough evidence to accept the alternate hypothesis concluding that there has not been a statistically significant difference between the Conventional Pink and Conventional blue module; clear and superslick module; superslick and Conventional blue module. The results for the Wilcoxon signed rank, a non parametric test, were similar to the paired t-test results for the four pairs namely Clear – Conventional Blue, Conventional Pink – Superslick, Clear – Conventional Pink and Clear – Superslick where the results showed that p-value of the test was close to zero for the first three pairs and 0.83 for Clear-Superslick pair. This implied that there was significant difference between the median of the modules in the first three pairs and there was not enough evidence to reject the null hypothesis which states that there was no significant difference between the median of clear and superslick module. However, the results for the Wilcoxon signed rank were different to the paired t-test results for the two pairs namely Conventional Blue-Conventional Pink and Conventional Blue – Superslick where the results showed that p-value of the test was less than 0.05 at 95% confidence intervals and thus, it could be concluded that we had enough evidence to accept the alternate hypothesis which states that there has been a significant difference between the median of the modules in the these two pairs ( Table 4). The adherence of *Streptococcus*, *Staphylococcus* and *Aerobic Lactobacilli* to the different modules was studied in terms of absolute/ relative frequencies ( Table 2). All the respondents were found to have streptococci adhered to conventional pink, while only 97.5% had this bacterial association on conventional blue module. Relative frequency of adherence was lowest (80%) in case of clear modules. The *p*-value of the chi-square test was calculated as 0.005. At 95% confidence intervals, we have enough evidence to accept the alternate hypothesis. Thus, it could be concluded that there was statistically significant difference in streptococcal adherence amongst the various modules, as depicted in clustered bar chart presented (Fig. 2). Similar statistical evaluations were performed for *Staphylococcus* and *Aerobic Lactobacilli* and their *p*-value of chi- square test were calculated as 0.156 and 0.166 respectively ( Table 2). Thus, indicating a statistically insignificant difference in adherence of both the bacterial genera amongst different modules. ( Table 2; Fig. 2).

**Table 4:**
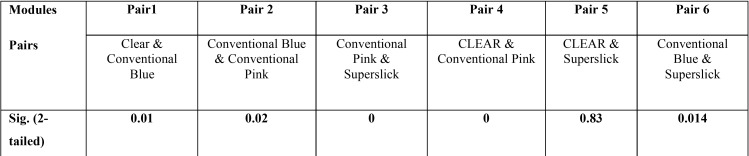
Wilcoxon Signed Rank test comparing the median of the four modules.

**Figure 3:**
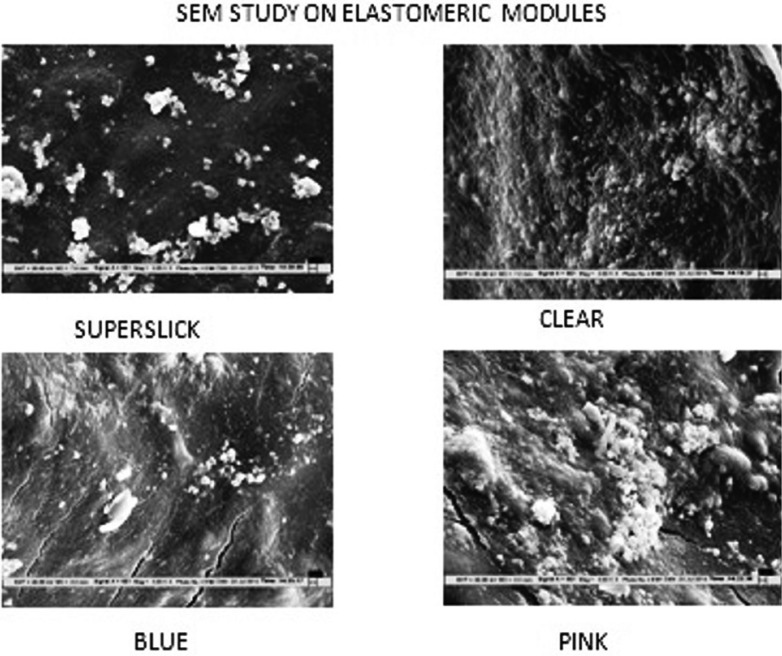
Scanning Electron Microscopy of four modules.

The scanning electromicrographs authenticated the bacterial adherence and fissures on to the modules in variable proportions (Fig. 3).

## Discussion

The present study attempted to evaluate the bacterial adherence to different elastomeric rings in orthodontic patients of 12-20 years age group who had almost similar oral hygiene practice, eating habits and no potential age-related differences in oral flora. Earlier, a number of studies have been carried out on bacterial colonization during orthodontic treatment and different ligation appliance and technique in almost similar age groups and approaches (2-4,7,9,10-20). The microbiological quality of saliva showed increased bacterial counts with presence of *Streptococcus*, *Staphylococcus* and *Aerobic Lactobacilli* in variable relative frequencies ranging from 80-100% at 6 week interval with the lowest frequency for *Aerobic Lactobacillus*. The increased bacterial counts and variable microbial population could be attributed to the increased areas of food accumulation afforded by components of the fixed orthodontic appliance and natural biological changes that might cause microbial assortment in oral cavity. The increased bacterial counts in saliva have earlier been also reported in response to orthodontic appliances. (Owen, 2008). The lowest frequency for *Aerobic Lactobacilli* reflected varied oral environment within the study samples. Earlier also, anaerobic *Lactobaciili* were found to increase in a ratio of 13:1 during the course of orthodontic treatment, keeping the probability of low prevalence for aerobic genera (4). Further, the intermodular differences in bacterial colonization were also evaluated.

Clear - Conventional Blue: The results showed a statistically significant difference between the bacterial counts on these two modules. The mean bacterial count on the Conventional blue placed at the lower right quadrant was higher than that of the counts on clear module placed at the upper left quadrant, by 24.09 X105 CFU/ml., indicating lesser bacterial colonization on the clear module. *Streptococcus*, *Staphylococcus* and *Lactobacilus spp.* were associated with both the modules in varying proportions, with higher relative frequencies (ranging from 3 to 17.5% higher) on conventional blue module. The difference in bacterial counts could be attributed to the brushing pattern followed by right handed person brushing with vigor and dexterity on the upper left quadrant. Moreover, SEM studies showed that the clear module had less fissures and surface roughness as compared to the blue module, thus causing lowered bacterial colonization. Earlier, higher microbial adherence has been seen on elastomeric ligatures as compared to steel (4,7).

Conventional Pink-Conventional Blue: There was hardly any significant difference in inter modular bacterial counts. *Streptococci*, *Staphylococcus* and *Aerobic Lactobacilli* were present on both the modules in all subjects. The heavy bacterial colonization on both these colored elastomers could be due to the suitable substratum for bacteria provided by morphological changes and strechability in the elastomers on incorporation of fillers in the form of pigments such as azo pigments, phthalocyanine pigments, nitro or nitroso pigments etc, to strengthen the materials, during the manufacturing process (21-23). The filler particles in polymer structure are said to have a large modulus than the surrounding structure, and consequently fail to extend to the same level as the remaining material, thus causing altered morphology and strechability of the elastomer. Moreover, dispersion of the pigment in the elastomer with the help of a master batch, such as polyethylene and polypropylene, a high-molecular substance could also lead to degradation or development of fissures in the elastomer. Literature cites that the primary structural influences on Glass temperature (Tg) are the composition, the steric interferences, and the types of bonding present for the molecule and that these factors influence molecular motion (21-23). Similar effects could be perceived on addition of pigments and fillers in coloured elatomers, thereby altering the clinical behavior of colored elastomers. Various studies corroborate the probable influences of these factors (24-28).

Superslick-Conventional Pink: The study showed statistically significant inter modular difference in bacterial counts. Aherence of *Streptococcus*, *Staphylococcus* and *Lactobacilus* on the Superslick module was 12.5%, 15% and 25% lower respectively as compared to Conventional pink. This could be due to the coating of superslick module with covalently bonded metafasix, claimed to reduce the friction of ligation by 60% due to hydrogel-polymer coating that renders the elastomeric surface as smooth and sliding on moistening. Earlier, hydrogel material resisted bacterial adhesion levels by 90% in six strains of bacteria (29). Less surface degradation of superslick rings in oral environment and greater stress concentration of non-coated elastomeric ligatures has also been reported (30). Our findings are consistent with the earlier findings in this regard (5,15).

Clear-Conventional Pink: The results indicated a statistically significant difference in bacterial colonization on Clear and Conventional pink modules. Bacterial adherence on the clear modules in upper left quadrant was lesser as compared to the Conventional Pink modules placed in the lower left quadrant. The scanning electron micrographs showed presence of more fissures and cracks on the surface of the Pink pigmented elastomeric module. Thus, said difference could be attributed to brushing patterns and incorporation of fillers in Pink modules as discussed.

Clear - Superslick: The bacteria adhered to the two modules in varying proportions, however, no statistically significant difference in bacterial colonization was observed. The adherence of *Streptococcus*, *Staphylococcus* and *Aerobic Lactobacilli* to clear module placed in the upper left quadrant was lesser by 7.5%, 5% and 12.5% respectively as compared to the Superslick module placed in the upper right quadrant. This could be to the result of immaculate brushing on the upper left quadrant by a right handed operator.

Superslick – Conventional Blue: A significant difference between the median of the modules in the Superslick modules placed in the upper right quadrant and Conventional blue module placed in the lower right quadrant was noticed. Moreover the bacterial adherence of the *Streptococcus spp*. *Staphylococcus* and *Aerobic Lactobacilli* was 10%, 5% and 15% lesser respectively on Superslick module as compared to the conventional blue module. As discussed earlier, conventional blue module might have altered structural morphology owing to the addition of fillers in the form of pigments making the surface rough, and creating niches, thereby giving an opportunity for the bacteria to adhere to the surface.

## Conclusions

Variable bacterial adherence with subsequent intermodular differences in colonization of *Streptoccccus*, *Saphylococcus* and *Lactobacillus* to four different ligatures was observed during orthodontic treatment, which could lead to poor oral health and unwanted pathologies. Thus, the colour dependent colonization of orthodontic ligatures could be an indicator of bacterial biofilm forming potential based on surface chemistries and a clinically efficacious tool to redisgn conventional and modified elastomeric rings as orthodontic ligation accessories. However, the said area need to be explored further.
